# The Importance of Connexin 43 in Enamel Development and Mineralization

**DOI:** 10.3389/fphys.2018.00750

**Published:** 2018-06-26

**Authors:** Sali Al-Ansari, Rozita Jalali, Lilian I. Plotkin, Antonius L. J. J. Bronckers, Pamela DenBesten, Yan Zhang, Judith E. Raber-Durlacher, Jan de Lange, Frederik R. Rozema

**Affiliations:** ^1^Department of Oral Medicine, Academic Center for Dentistry, Amsterdam, Netherlands; ^2^Department of Orofacial Sciences, School of Dentistry, University of California, San Francisco, San Francisco, CA, United States; ^3^Department of Anatomy and Cell Biology, Indiana University School of Medicine, Indianapolis, IN, United States; ^4^Roudebush Veterans Administration Medical Center, Indianapolis, IN, United States; ^5^Indiana Center for Musculoskeletal Health, Indianapolis, IN, United States; ^6^Department of Oral Cell Biology, Academic Center for Dentistry, Amsterdam, Netherlands; ^7^Department of Oral and Maxillofacial Surgery, Amsterdam Medical Centre, University of Amsterdam, Amsterdam, Netherlands

**Keywords:** enamel, hypomineralization, microCT, mineralized tissue development, ameloblast, connexin 43

## Abstract

During enamel development, formation of hydroxyapatite crystals and regulation of pH in the enamel matrix require massive transport of ions. Both ameloblasts and adjacent dental epithelial cells in the stellate reticulum co-express several transmembrane cotransporters/ion-exchangers for transport of ions across plasma membranes. Gap junctions (GJs) enable intercellular exchanges of ions between neighboring cells. This suggests that the ameloblasts and other cell layers of the enamel organ, form a functional unit. During the bell stage of tooth formation, the non-ameloblast dental epithelium highly expresses the Na-K-Cl cotransporter (Nkcc1). *Nkcc1*-null mice are associated with enamel hypomineralization and increased expression of GJ protein connexin 43 (Cx43), suggesting that reduced ion transport in the *Nkcc1*-null mouse is in part compensated by increased intercellular ion transport through GJs. To understand the role of GJs in ion transport and its effect on pH regulation, we examined in a mouse strain in which Cx43 was ablated selectively in DMP1 expressing cells (Cx43^flox/flox^ mice crossed with DMP1-8kb-Cre mice), including ameloblasts. Micro-CT analysis showed that the mineral density at late maturation stage incisal enamel of the *Cx43*-null mice was 10% less than in controls, whereas that in dentin was unchanged. Maturation stage ameloblasts of mice lacking the pH regulating sodium/bicarbonate transporter NBCe1 (*Nbce1-*null), or chloride channel Cftr (*Cftr-*null) were found to have increased Cx43-immunostaining. These results support the possibility that GJs in the ameloblast–papillary complex at the maturation stage contribute to ion transport by enabling passage of ions directly from cells of the papillary layer into ameloblast layer. Increasing the number of GJs may partly compensate the reduction of ion-cotransporters and ion exchangers in dental epithelium.

## Introduction

Dental enamel is the hardest substance in the human body. Enamel mineralization is an active process, regulated by enamel organ cells, including ameloblasts, stratum intermedium cells, stellate reticulum, and the papillary layer. Secretory stage ameloblasts differentiate from pre-ameloblasts to synthesize and secrete proteins into the matrix space to form the full thickness of matrix, into which long thin hydroxyapatite crystals grow. At the end of the secretion stage, capillaries invaginate the stellate reticulum layer, which overlies ameloblasts, to form the papillary layer, which is rich in capillaries. After a short transition stage there is a rapid increase in mineral deposition into the matrix, which is the start of the maturation stage (Nanci, 2012). During the maturation stage enamel proteins are removed from the enamel space and calcium and other ions necessary for enamel formation are deposited (Smith, 1998). The differentiating enamel organ is thought to be solely responsible for all these activities (Elwood and Bernstein, 1968). At the maturation stage, expression patterns of the ion exchangers of SLC4A4 (NBCe1) and Na^+^-K^+^-ATPase (Lacruz et al., 2010b; Jalali et al., 2014; Wen et al., 2014), in both ameloblasts and papillary cells, suggest that both layers form a functional unit to regulate the hemostasis of ion sodium and potassium exchange to direct matrix mineralization. Changes in the structure of the papillary layer between early and late maturation stages may reflect changing rates of calcification or of resorption of enamel proteins.

Ameloblasts and papillary cells are likely to communicate with each other through cell–cell junctions. In general, cell–cell junctions, including adherents junctions, tight junctions, and gap junctions (GJs) participate in cell–cell communication. Among those cell–cell junctions, GJs are the only junction type that allows direct intercellular ion exchange. Connexins are trans-membrane Connexins (Cxs) proteins that can form intercellular channels to allow ion and small molecule (<1.5 kDa) exchange between cells (Evans and Martin, 2002). Twenty-one different Cx isoforms have been identified in humans and twenty Cx isoforms in mice (Söhl and Willecke, 2004). Cx43 is highly expressed in rodent incisors, molars and the cells forming the human tooth during development, including the ameloblasts, stratum intermedium, stellate reticulum, papillary layer, and odontoblasts (João and Arana-Chavez, 2003, 2004; Toth et al., 2010).

Mutations in connexin genes lead to alterations in important biological functions of GJ channels and hemi channels, disturbing intercellular communication (Laird, 2006). Mutations in human Cx43 results in oculodentodigital dysplasia (ODDD), an autosomal-dominant disorder characterized by anomalies of face, eyes, limbs, and teeth (Paznekas et al., 2003). The most common symptoms are enamel hypoplasia, microdontia, micro-cornea and craniofacial, skeletal and skin alterations (Weintraub et al., 1975; Gladwin et al., 1997; Paznekas et al., 2003, 2009). Mice lacking Cx43 die within hours after birth because of cardiac malformations precluding the study of the adult skeleton (Langille et al., 1995). Dominant negative G60S mutants of Cx43 (Flenniken et al., 2005) mice have an altered enamel organ morphology and enamel hypoplasia (Toth et al., 2010). Recently, we found that expression of Cx43 was enhanced in enamel organ of mice lacking *Nkcc1* suggesting that increased numbers of GJs may compensate for deficient ion transport by transmembrane cotransporters, including downregulation of Nkcc1 (Jalali et al., 2017).

In this study we tested the hypothesis that GJs contribute to enamel formation by enabling passage of ions from papillary layer into ameloblasts. We examined this by comparing the expression of *CX43* protein in the enamel organs of Cx43 deficient in ameloblasts (*Cx43^flox/flox;DMP1-Cre^*). Our further characterization of Cx43 in enamel organs of *Cftr* and *Nbce1* null mice, both of which transport ions involved in ameloblast regulation of pH on the forming extracellular enamel matrix (Bronckers et al., 2010; Jalali et al., 2014) supported our hypothesis by showing a compensatory upregulation of Cx43 in these mouse models.

## Materials and Methods

### Tissues

One-month-old male mice lacking Cx43 cells expressing DPM1 (Cx43^flox/flox;DMP1-8kb-Cre^, *N* = 4) and control littermates (Cx43^flox/flox^, *N* = 5) were generated and genotyped as previously described (Bivi et al., 2012). Other mouse strains and procedures for this study have been described before (Bronckers et al., 2010; Jalali et al., 2014). For each genotypic mouse strain, teeth, and enamel organs from three wild type mice and three null mutant mice were analyzed and compared. All animal procedures were approved by the Committee for Animal Care using standards for treatment of animals (University of Amsterdam), the Animal Care and Use Committee of the National Institute of Dental and Craniofacial Research, National Institutes of Health (ASP 13-686), and the Institutional Animal Care and Use Committee of Indiana University School of Medicine.

### Histological Procedures

Upper and lower jaws of wild type, Cx43^flox/flox;DMP1-8kb-Cre^, Cx43^flox/flox^, *Nbce1* null, and *Cftr-* null mice were fixed by immersion in 5% paraformaldehyde in 0.1 M phosphate buffer pH 7.3. Hemimandibles were then decalcified in 4% EDTA, pH 7.3 for 2 weeks at 4°C and processed into paraffin. Sagittal serial sections in thickness of 5–7 μm were prepared and mounted on polylysine coated glass slides.

### Immunohistochemistry

Dewaxed sections were rinsed in phosphate buffered saline (PBS) and subjected to antigen retrieval in 10 mM citrate buffer (pH 6.0) either at 60°C overnight, or for 20 min at 95°C in a microwave oven. After antigen retrieval, endogenous peroxidase was blocked with a peroxidase block solution (Envision kit, Dakocytomation) for 5 min. Sections were washed in TBST (3x). Non-specific staining was blocked for 30 min with 2% BSA. Sections were then incubated overnight at 4°C with rabbit anti-Connexin-43 (Cx43) (Abcam, Catalog No. Ab11370). Matched non-immune IgG (1:200 to 1:300) or normal serum (same concentration as primary antibodies) served as controls. After overnight incubation at 4°C with primary antibodies, sections were washed three times in TBS and incubated with goat anti-rabbit IgG antibodies conjugated with peroxidase (EnVision Kits) for 1 h at room temperature and counterstained with hematoxylin. Otherwise, rabbit anti-Cx43 was visualized by goat anti-rabbit Alexa Fluor 488 (5 μg/mL; Invitrogen) after 1 h incubation at room temperature and counterstained with DAPI (Vector Laboratories, Burlingame, CA, United States). Immunohistochemistry images were acquired with Leica EL6000 or Axio Zoom V16 microscope.

### Microcomputed Tomography (microCT)

To determine the degree of mineral content, hemi-mandibles from Cx43^flox/flox;DMP1-8kb-Cre^, Cx43^flox/flox^ mice were scanned at a resolution of 8 μm voxel using a μCT-40 high resolution scanner (Scanco Medical, AG, Bassersdorf, Switzerland). Mineral density was determined at sequential stages of development. Cross-sectional virtual images were collected from the most developed (incisor tip) to the least developed (cervical) area. The most incisal slice containing the most mineralized enamel was identified visually. Measurements were made at 500 μm intervals (50 slices at 10 μm interval) and slices at the same developmental stage from three mice per group were averaged and plotted as function of stage (slice number). Independent *t*-test was used to compare the groups. Statistical significance was set at *p* < 0.05 level.

### Western Blotting

From freeze-dried wild type and *Cftr* null mutant mandibular incisors early maturation stage enamel organs were micro dissected incisally based on a reference line projected between the first and second molars (M1 and M2), to separately dissect secretory and maturation stage ameloblasts, as described before (Schmitz et al., 2014). The apical, secretory, half of the enamel organ was placed in SDS loading buffer (from Nucleospin Triprep kit, Macherey-Nagel, supplied by Bioke, Leiden, Netherlands) and protein was measured using the BCA protein assay (Bio-Rad, Hercules, CA, United States). Twenty 20 μg of enamel organ denatured protein and 10 μg of molecular weight markers (Novex^®^ Sharp Pre-stained Protein Standard (# LC5800) or SeeBlue^®^ Plus2 Pre-stained Protein Standard (#LC5925) were subjected to electrophoresis in a 3–8% Tris acetate Nupage gel with Tris acetate running buffer for 60 min at 150 V or 4–12% Bis-Tris Protein Gels with MOPS buffer for 35 min at 200 V. Subsequent electro blotted by an iBlot device (Invitrogen) on nitrocellulose membrane was performed according to the manufacturer’s instructions. Membranes were blocked with BSA 2% for 1 h at room temperature and incubated overnight with the primary antibodies. Blots were then washed three times in TBST and incubated in with IRDye secondary antibodies (LI-COR). Visualization and quantification was carried out with the LI-COR Odyssey scanner and software (LI-COR Biosciences). Red color (for actin) was detected at 680 nm wavelength and a green color (other primary antibodies and tubulin) was detected at 800 nm wavelength. Quantification was performed using Odyssey software. Intensity values of the bands were normalized for actin or tubulin and expressed as percentage of wild type (100%). These primary antibodies were used: rabbit anti-Cx43 (Abcam, see above), and anti-tubulin rabbit antibody (ab, 59680). Secondary antibodies: IRDye 800CW conjugated goat anti-rabbit IgG (H+L) highly cross-adsorbed (LI-COR; Product No. 926-32211). Dilutions: anti-tubulin (1:1000); anti-connexin 43 (1:250); secondary antibodies [antibodies (1:10000)].

### Quantifications in Enamel Organ

Paraffin embedded incisor sections were dewaxed in xylene, rehydrated in descending grades of ethanol and stained with 1% hematoxylin (15 min) and eosin (5 min). Sections were dehydrated in ascending grades of ethanol and xylene and mounted in Depex mounting medium. The enamel organs were imaged under 40x objective lenses. Images of secretory and maturation stage of enamel organ were selected based on the anatomical position in the tooth. The number of GJ plaques were counted as previously described (Toth et al., 2010). The investigator was blinded, and GJs (defined as a discernable Cx43-labeled bright fluorescent structure of at least 0.5 μm in length, seen in membranes of adjacent cells) were counted per field of view and normalized to the number of the cells in the area (number of DAPI-stained nuclei) as described before. Stratum intermedium (SI), papillary layer (PL), and ameloblasts were counted separately and combined for a total enamel organ assessment. Statistical analysis was performed by independent *t*-test (*p* < 0.05).

## Results

### Connexin 43 Expression Is Important for Enamel Organ Function and Enamel Mineralization

**FIGURE 1:**
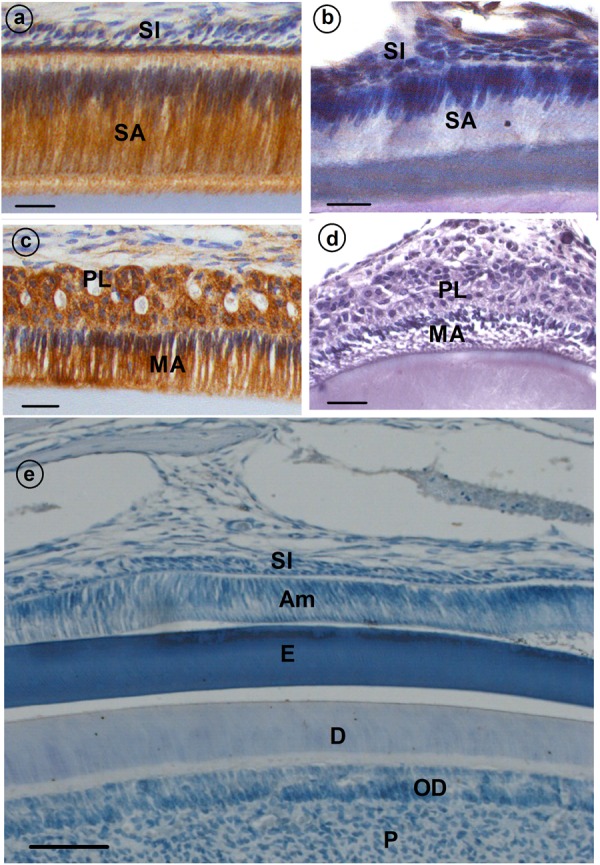
Immunolocalization of gap junction (GJ) protein (CX43) **(a–d)** in developing mouse teeth. Secretory ameloblasts (SA; **a**) and maturation ameloblasts (MA; **c**) are positive for CX43; some staining is also presents in papillary layer (PL; **c**). **(b,d)** Cx43^flox/flox;DMP1-8kb-Cre^ incisor stained with anti-CX43. **(e)** A sagittal section through the incisor of a control mouse of Cx43fl/fl in which normal rabbit IgG was used as primary antibody instead of rabbit anti-Cx43 antibody. All the stainings have been tested in triplicate in three mice. SI, stratum intermedium; SA, secretory ameloblasts; E, enamel; MA, maturation ameloblasts; PL, papillary layer; P, pulp; OD, odontoblasts.

Histological assessment of enamel organs from Cx43^flox/flox^ control mice revealed a tall, columnar ameloblast cell layer in both the secretory and maturation stage of enamel formation (**Figures 1a,c**). However, both secretory and maturation stage ameloblasts from Cx43^flox/flox;DMP1-8kb-Cre^ mice revealed no evidence of cell polarity and the columnar profile of this epithelial cell layer was lost (**Figures 1b,d**; Cx43^flox/flox;DMP1-8kb-Cre^) and **Figure 1e** (Cx43^flox/flox^ stained with IgG serum antibody) shows the specificity of the antibody.

**FIGURE 2:**
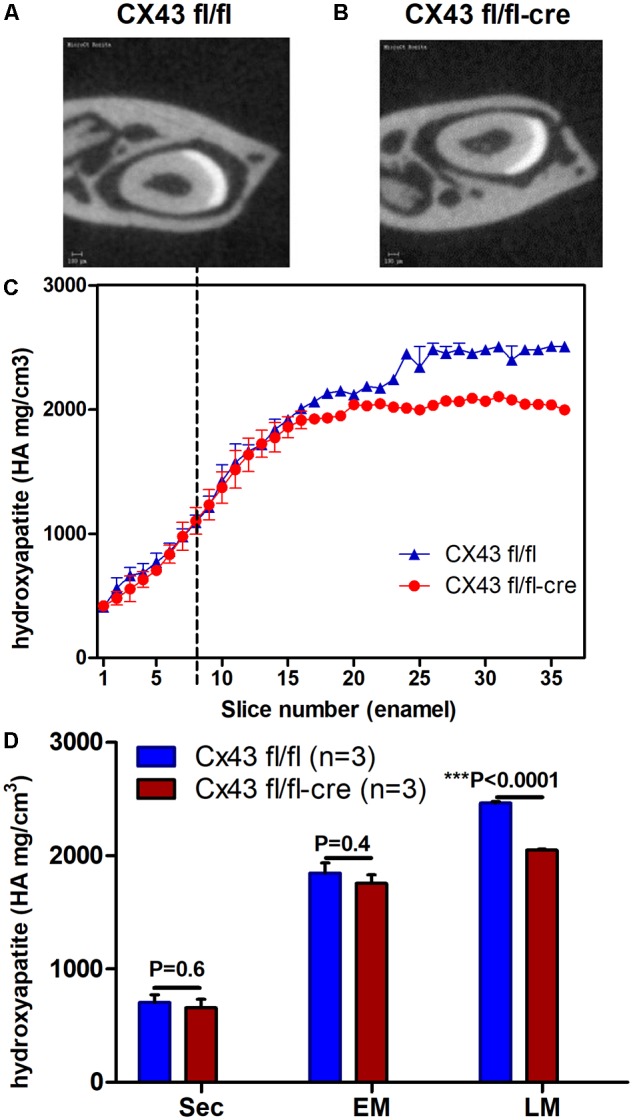
Lower mineral density of enamel in maturation stage ameloblasts of Cx43^flox/flox;DMP1-8kb-Cre^ **(A–D)** (*n* = 3) suggest the importance of CX43 for enamel mineralization. Mineral density measured by micro-CT plotted against slice numbers **(C)** (blue color represents Cx43*^fl/fl^* and red Cx43^flox/flox;DMP1-8kb-Cre^ lower incisor). Secretory stage starts from zero and maturation stage starts after dotted lines (*n* = 3 in each group). In **(D)** the bar graphs with the same color (blue and red) represent measurements of mineral density in different stages of amelogenesis (sec, secretory; EM, early maturation; LM, late maturation). Figures **(A,B)** show virtual cross-section of Cx43*^fl/fl^* and Cx43^flox/flox;DMP1-8kb-Cre^ lower incisor, respectively. The difference in dentin between **(A,B)** is most likely due to differences in angle of the section. The number of teeth per group is *N* = 3; error bar represents SE.

To investigate whether absence of CX43 in dental epithelium influences enamel mineralization micro-CT analysis was carried out on jaws of conditional *Cx43* ko mice as compared to controls. In upper incisors disruption of the CX43 gene reduced mineral density by ∼10% at late maturation (*p* < 0.0001) but not at secretory and early maturation phase (**Figures 2A–D**). The fact that in the mutant teeth enamel was hypomineralized at late maturation stage suggested that GJs are required to fully mineralize enamel at late maturation.

### Cx43 Expression Is Increased in *Nkcc1-null* and *Cftr* Null Mouse Enamel Organs

**FIGURE 3:**
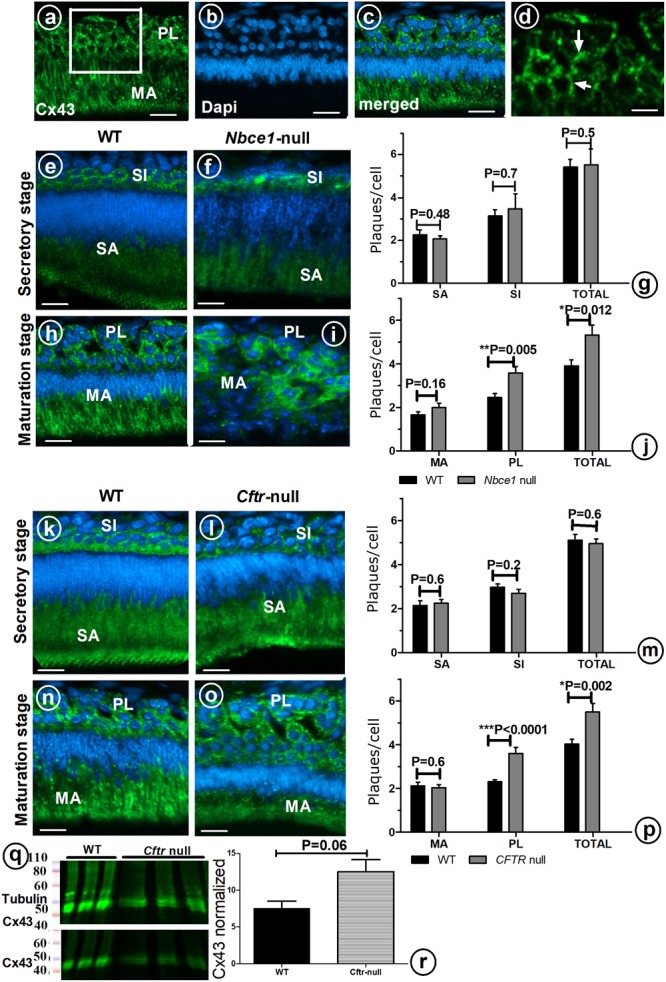
Enamel organ cells of wild type **(a–d,e,h,k,n)**, *Nbce1* null **(f,i)**, and *Cftr* null **(l,o)**. Upper panel shows anti-Cx43 (green) **(a)**, DAPI nuclear staining (blue) **(b)** and superimposed a on b **(c)**. **(d)** At high magnification discrete plaques are apparent which were counted per cell type (Indicate a few with arrow). Bar diagram **(g,j,m,p)** presents plaque counts in various cell layers. **(q)** Enamel organ extracts of *Cftr* null mice stained with anti-Cx43 and normalized to tubulin. **(r)** Bar diagram presents the amount of Cx43 protein, although increased in *Cftr* null mice, the difference did not reach statistical significance (*n* = 3 mice, for each group). SI, stratum intermedium; SA, secretory ameloblasts; MA, maturation ameloblasts; PL, papillary layer. Bar = 50 μm.

To examine the possible role of GJs in directing the transport of ions from the papillary layer to ameloblasts, tissue sections of mouse mutants with hypomineralized enamel as result from disruption in local pH regulation (*Cftr-* null and *Nbce1-* null mice) were immunostained for Cx43. In wild type mouse *secretory* ameloblasts and stratum intermedium strong positive staining for Cx43 was seen in the basolateral plasma membranes; weaker staining was observed in stellate reticulum cells located between the stratum intermedium and outer enamel epithelium (**Figures 3e,k**). Wild type *maturation* ameloblasts and papillary layer stained strongly for Cx43 (**Figures 3a–d,h,n**).

In *Nbce1* null mice (**Figures 3f,i**) and *Cftr* null mice (**Figures 3l,o**) the distribution pattern of immunostaining for Cx43 in enamel organs was largely similar as in wild type mice. Protein expression levels of Cx43 in enamel organs by *Western blotting* showed that Cx43 was increased by 65% (*p* = 0.06) in *Cftr* null mice (**Figures 3q,r**).

Immunohistochemical staining with anti-Cx43 showed that in both *Nbce1* null and *Cftr* null mice the total number of GJs (number of fluorescent Cx43 positive plaques in the enamel organ), was not different from wild type controls in secretory stage (**Figures 3g,j,m,p**) but had increased by in maturation stage but significantly increased in the *papillary layer* in maturation stage (*Cftr null* mice, *p* < 0.0001; for *Nbce1* null mice *p* = 0.005 respectively; **Figures 3g,j,m,p**).

## Discussion

These results, which show that that differentiating ameloblasts and all cells of the enamel organ, i.e., the stellate reticulum and stratum intermedium express Cx43, and are consistent with previous reports (João and Arana-Chavez, 2003; Toth et al., 2010). However, our results, which show that the loss of Cx43 GJs only affect enamel mineralization at the late stage of enamel formation, do not support the proposal by Toth et al. (2010) that Cx43 GJs may mediate protein deposition required for final matrix mineralization.

Cx43^flox/flox;DMP1-8kb-Cre^ mice used in this study, have Cx43 conditionally deleted in cells expressing dentin matrix protein 1 (DMP1). Though DMP1 is expressed by both ameloblasts and ondotoblasts (D’souza et al., 1997; Martinez et al., 2009), in our study the loss of Cx43 did not have an obvious effect on odontoblast differentiation and dentin mineralization (data not shown), suggesting that morphologically changes in differentiating ameloblasts are specifically due to the loss of Cx43 in ameloblasts. Secretory stage ameloblast differentiation occurs in response to signaling that occur as dentin mineralization is first initiated (Bartlett, 2013), and our finding that secretory ameloblasts of the null mice polarize and have similar morphology to wild type mice, further supports the use of this mouse model to investigate the role of Cx43 GJs in ameloblast mediated enamel bio-mineralization.

Micro-CT analysis showed and effect on mineralization only at the maturation stage of enamel formation in Cx43^flox/flox;DMP1-8kb-Cre^ mice, suggesting an important function for GJs in later enamel matrix mineralization. To test the hypothesis that Cx43 GJs direct transcellular transport of ions between papillary layer cells and ameloblasts to allow final matrix mineralization, we measured the amount of Cx43 in western blots and counted the Cx43 immunopositive plaques in mouse mutants with established hypomineralization of enamel during maturation stage (*Cftr* null mice and *Nbce1* null mice) (Bronckers et al., 2010; Jalali et al., 2014). Western blots showed an increase of Cx43 protein in enamel organs of *Cftr* null mutant; immunohistochemically more Cx43 plaques were counted in papillary layer of *Cftr* null and *Nbce1* null mice than in wild type controls. These findings suggest that an increase of the number of GJs can occur to increase ion transport through dental epithelium intracellularly when ion exchangers do not function properly.

Ion exchangers are required to regulate pH resulting from the release of hydrogen ions that acidify the enamel matrix as hydroxyapatite mineral is formed (Lacruz et al., 2010a, 2012, 2013). At earlier stages of enamel formation the release of hydrogen ions is buffered by amelogenins in the enamel matrix (Guo et al., 2015). Up-regulation of amelogenin in Gja1Jrt/þ (in Gja1Jrt/þ mice the expression of Cx43 is reduced), ameloblasts suggests that compensation of amelogenins in the absence of GJ proteins may responsible for enamel matrix mineralization at secretory stage of amelogenesis. However, in the maturation stage, where we saw hypomineralization in the Cx43^flox/flox;DMP1-8kb-Cre^ mice, the regulation of matrix acidification by ion transporter(s)/channel(s)/exchanger(s) is crucial.

Taken together, the results of this study support the importance of GJs in ion transport through the enamel organ epithelium. Furthermore, these results support the model that the cells of the papillary layer and ameloblasts form a functional unit to transport ions to the ameloblasts for pH regulation of the final enamel matrix mineralization.

## Author Contributions

SA-A and RJ: implemented the experiments and analysis and wrote the manuscript. AB, PD, YZ, JR-D, JdL, and FR: contributed to the design and implementation of the research, manuscript revision, and data analysis. LP: contributed to the design and implementation of the research, manuscript revision, and data analysis, provided the CX43 mice.

## Conflict of Interest Statement

The authors declare that the research was conducted in the absence of any commercial or financial relationships that could be construed as a potential conflict of interest.
